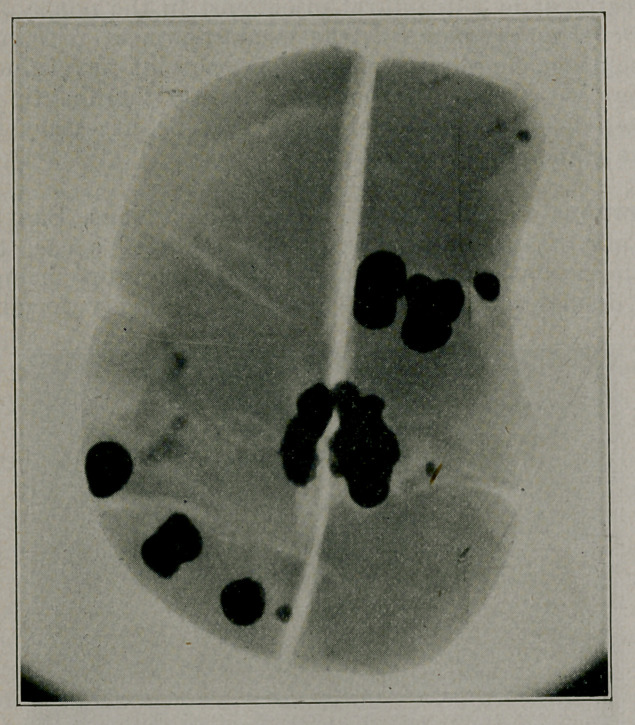# Concretions of the Spleen

**Published:** 1916-08

**Authors:** 


**Concretions of the Spleen,** Benjamin Jablons, San Francisco, Calif. State Jour., Mch. Sailer reported a case to the Philadelphia Pathological Society, 1897, the stone being 1/2 c.m. in diameter, with smooth surface, probably forming in an old thrombus. It was found at necropsy in an old man, cause of death not stated. Orth had alluded to the subject without details. Dufour, Bull. et Mem. de la Soc. Med. des Hop. de Paris, 1903, reported a multiple case, also found at necropsy in an old man, who had had chronic bronchitis and emphysema and who showed lesions of healed tuberculosis. The spleen appeared normal but six calculi were found on section, about the size of cherry pits, and surrounded by a sort of capsule. Ca, Mg and Na carbonate and phosphate were found chemically. There was also a similar stone, the size of a pin's head in the liver. Defour considered them as probably calcified tubercles, possibly hydatid cysts. These are the only cases that Jablons could find in the literature.

Jablons's case occurred in a man with ulcerating cancer of the penis with metastases to the inguinal glands. The history was not significant except for yellow fever 40 years earlier. At necropsy, extensive arteriosclerosis, emphysema and chronic bronchitis were found. There was no evidence of tuberculosis except possibly a plastic pleurisy. The spleen was somewhat enlarged, 105 grams, 7.5x6x2 c.m., and there had been some perisplenitis. The multiple calculi are shown photographically and by a radiograph of the spleen. The author's explanation is that there was first an interstitial splenitis, with dilatation of veins, thrombi and ultimately calcification. (See photomicrograph). Cuts by courtesy of author and editor.

## Figures and Tables

**Figure f1:**
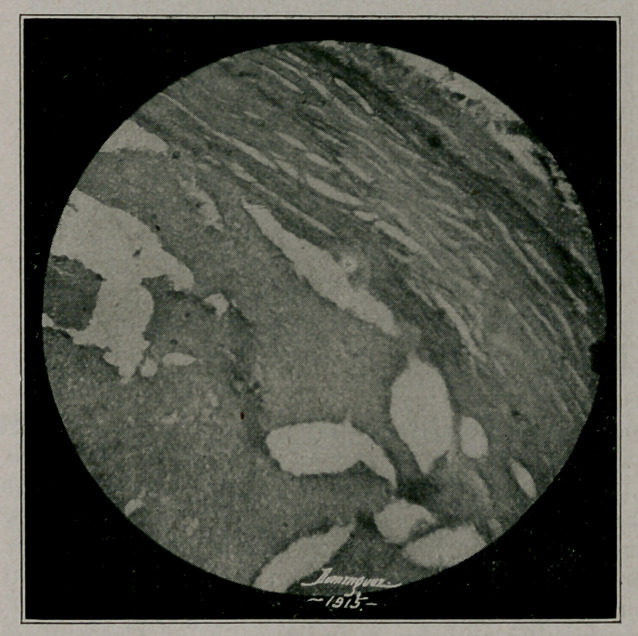


**Figure f2:**